# Preparedness of CT technicians and radiographers for severe contrast media reactions: knowledge gaps and training needs

**DOI:** 10.3389/fradi.2026.1940155

**Published:** 2026-09-10

**Authors:** Haneen E. Abuanzeh, Ayman M. Al-Qaaneh

**Affiliations:** Faculty of Allied Medical Sciences, Al-Balqa Applied University (BAU), Al-Salt, Jordan

**Keywords:** computed tomography, contrast media reactions, patient safety, radiographers, radiologic technologists

## Abstract

**Background:**

Severe contrast media reactions during computed tomography (CT) are rare but potentially life-threatening and require rapid recognition and coordinated emergency response. CT technicians and radiographers are often frontline professionals during contrast-enhanced CT, yet their preparedness in Jordan remains underexplored.

**Objective:**

To assess knowledge of severe contrast media reactions among CT technicians and radiographers in Jordan and identify factors associated with total knowledge scores.

**Methods:**

A descriptive cross-sectional study was conducted using an online questionnaire adapted from a previously published tool. The questionnaire assessed five domains: general knowledge of contrast media, risk factors, precautions, recognition of severe reactions, and management. Scores were converted to percentages. Data were analyzed using descriptive statistics, Mann–Whitney U tests, Kruskal–Wallis tests, Spearman correlation, and multivariable linear regression.

**Results:**

The analysis included 314 participants. The median total knowledge score was 76.3% (IQR: 55.3–84.2). General knowledge showed the highest median score (100%, IQR: 80–100), whereas risk factor knowledge was the lowest-performing domain (65%, IQR: 50–80). Radiographers achieved higher total knowledge scores than technicians (78.9% vs. 71.0%, *p* = 0.002). Total knowledge also differed significantly across educational levels (*p* = 0.043). In multivariable regression, total knowledge was independently associated with gender, age group, master's-level education, professional role, experience >25 years, and self-reported confidence.

**Conclusion:**

CT technicians and radiographers in Jordan demonstrated moderate-to-high overall knowledge of severe contrast media reactions, but important gaps were evident, particularly in risk factor recognition. Structured, recurrent, competency-based training programs focusing on risk assessment, reaction recognition, emergency management, and simulation-based practice are needed to strengthen preparedness and patient safety during contrast-enhanced CT.

## Introduction

Intravenous contrast media (IVCM) have significantly enhanced the diagnostic capability of computed tomography (CT), enabling the generation of highly detailed and accurate images that improve clinical decision-making and patient care (1). These advancements are particularly important in the diagnosis of complex conditions such as cardiovascular diseases and malignancies, where early detection is essential for timely intervention and improved clinical outcomes (2).

Despite their diagnostic benefits, the administration of contrast media may be associated with adverse reactions. Historically, early hyperosmolar contrast agents were associated with relatively high rates of allergic and physiologic reactions, reaching up to 15% in some reports (3). To standardize the classification of these reactions, the American College of Radiology (ACR) categorizes contrast media reactions into two main types: physiologic reactions and allergic-like reactions, each further subdivided into mild, moderate, and severe forms. Physiologic reactions may include vasovagal responses, pain at the injection site, infusion-related sensations, and neurologic manifestations, some of which can be life-threatening if not promptly managed (4).

Clinically, contrast media reactions are generally classified according to their severity. Mild reactions typically include symptoms such as nausea, vomiting, urticaria, and localized discomfort. Moderate reactions may involve bronchospasm, hypertension, or diffuse erythema. Severe reactions, although rare, can include pulmonary edema, cardiac arrhythmias, anaphylactic shock, circulatory collapse, and loss of consciousness (5). The underlying mechanisms of these reactions vary; mild and moderate reactions are often associated with histamine-mediated hypersensitivity responses, whereas severe reactions may involve endothelial injury, direct myocardial depression, or severe anaphylactoid responses that require immediate medical intervention (6).

With the introduction of modern low-osmolar contrast agents, the incidence of severe adverse reactions has decreased substantially and is estimated to occur in approximately 0.1%–0.2% of cases. Nevertheless, the potential for life-threatening complications underscores the critical importance of rapid recognition and effective management to ensure patient safety during CT procedures (7).

Several international professional bodies -including the American College of Radiology (ACR), the Royal College of Radiologists (RCR), the European Society of Urogenital Radiology (ESUR), and the Royal Australian and New Zealand College of Radiologists (RANZCR)- have developed guidelines outlining the roles and responsibilities involved in the management of contrast media reactions (8–11). Although radiologists generally assume primary responsibility for clinical decision-making during severe reactions, CT radiographers and technicians play a crucial frontline role in patient monitoring, early recognition of adverse reactions, and initiation of emergency response protocols.

Across these guidelines, several common recommendations are emphasized, including ensuring emergency preparedness, continuous patient monitoring following contrast administration, and identifying individuals at increased risk for adverse reactions, such as those with previous contrast reactions, asthma, or renal impairment. However, variations exist among these guidelines regarding premedication protocols, the level of autonomy granted to radiographers in managing contrast reactions, and the distribution of responsibilities during emergency situations (8, 9). For example, the RANZCR provides more explicit competency requirements for radiographers in administering emergency medications under supervision, whereas the ACR and ESUR emphasize the availability of resuscitation equipment and active patient monitoring by radiographers (12).

Evidence from the literature suggests that radiographers' preparedness to manage severe contrast media reactions varies considerably across regions. Studies conducted in Southeast Asia and the Middle East have identified substantial differences in the implementation of international contrast safety guidelines, particularly regarding emergency preparedness and training (13). Similarly, a study conducted in Lithuania demonstrated that radiographers with greater clinical experience exhibited higher levels of competence and confidence in managing contrast reactions, indicating that practical experience plays a key role in skill development (14).

Comparable findings have been reported in Saudi Arabia, where senior radiographers demonstrated significantly higher knowledge scores in managing severe contrast media reactions (SCMR) compared with junior staff. In addition, radiographers who received training abroad showed greater proficiency, possibly reflecting differences in educational curricula, clinical exposure, and the availability of structured emergency training programs (6).

These findings highlight the importance of implementing structured training programs aligned with international best practices to ensure that all radiographers possess the competencies required to effectively manage SCMR. A major limitation of many current training models is that they primarily focus on radiologists and other physicians, with comparatively limited attention given to radiographers. The absence of standardized training programs specifically designed for radiographers contributes to variability in preparedness, and in some institutions formal protocols for managing SCMR may be insufficient or absent altogether (13).

Therefore, this study aims to evaluate the ability of CT technicians and radiographers to recognize and effectively manage severe contrast media reactions. Additionally, the study seeks to assess the extent to which existing training programs and departmental policies prepare radiographers for contrast-related emergencies. By identifying key knowledge gaps and training deficiencies, this research aims to support the development of standardized educational programs that enhance radiographers' preparedness and promote patient safety in radiological practice while aligning with international best practices.

## Materials and methods

### Study design, participants, and ethical approval

A descriptive cross-sectional study was conducted using an online questionnaire to evaluate the ability of CT technicians and radiographers in Jordan to recognize and effectively manage severe contrast media reactions. In the Jordanian healthcare context, CT technicians/technologists are medical imaging professionals, typically trained in radiologic sciences at the diploma, bachelor's, or postgraduate level, who are responsible for patient preparation and positioning, technical performance of CT examinations, optimization of imaging parameters, radiation safety, and contrast administration when appropriately trained and authorized. They generally work according to institutional protocols and under the clinical supervision of a radiologist. In contrast, radiographers are physicians who have completed postgraduate specialist training in diagnostic radiology and are responsible for image interpretation, clinical decisions regarding contrast-enhanced examinations, assessment of patient-specific risks and contraindications, and the medical management of adverse contrast-media reactions. Data were collected between December 17, 2025, and February 6, 2026, using Google Forms. The survey was distributed through social media platforms as part of a digital recruitment campaign targeting a convenience sample of CT technicians and radiographers working in Jordan.

The estimated eligible professional population in Jordan was approximately 3,250, comprising approximately 2,800 CT technicians and 450 radiographers. Eligible participants were CT technicians and radiographers currently employed in governmental, military, or private hospitals or healthcare centers in Jordan. CT technicians were required to be currently working in CT or to have previous hands-on professional experience with CT modalities. CT technicians without previous CT experience were excluded. In addition, CT technicians and radiographers who were not currently employed were excluded. No formal *a priori* sample-size calculation or power analysis was performed. Recruitment was based on feasibility and accessibility during the study period, and approximately 370 eligible professionals were invited to participate.

To reduce duplicate responses, the survey settings allowed only one response per participant. Data were collected anonymously, and no personally identifiable information was included in the study dataset. Electronic informed consent was obtained from all participants before they proceeded to the survey questions. The questionnaire was written, validated, and administered in English. Ethical approval was obtained from the Institutional Review Board at Al-Balqa Applied University/Al-Salt (Approval No. 2026/2025/3/51).

## Study tool

The data collection instrument used in this study was adapted from a previously published questionnaire after formal permission was obtained from the original author(s) (6). The questionnaire was designed to assess the ability of CT technicians and radiographers to recognize and manage severe contrast media reactions.

The original questionnaire development process involved drafting and refining the instrument based on feedback from 15 experienced CT radiographers to improve its clarity, relevance, and applicability to clinical practice. The revised questionnaire was subsequently re-evaluated by the same group to confirm its final structure before official distribution.

For application in the Jordanian context, the adapted questionnaire underwent content review by a panel of three experts comprising an academician, a radiologist, and a senior CT technician. The panel assessed the appropriateness, relevance, and clarity of the questionnaire. The questionnaire was subsequently pilot-tested among nine participants from the target population. No concerns requiring modification were identified during pilot testing; therefore, the questionnaire was administered without further changes, and the pilot participants' responses were retained in the final analysis.

The questionnaire consisted of two sections. Section I included 13 items collecting demographic and professional information from CT technicians and radiographers. Section II included five domains assessing knowledge related to contrast media and associated adverse reactions. These domains were: general knowledge of contrast media, assessed using five true/false questions; knowledge of risk factors associated with severe adverse reactions, assessed using 10 questions with “risk factor,” “not a risk factor,” and “don't know” response options; knowledge of precautionary measures to reduce the risk of severe reactions, assessed using five true/false questions; recognition of severe contrast media reactions, assessed using 10 questions with “severe,” “non-severe,” and “don't know” response options; and knowledge of the management of severe adverse contrast media reactions, assessed using eight true/false questions.

Each correct answer was assigned one point, while incorrect and “don't know” responses were assigned zero points. The total possible raw score was 38. Raw scores were then standardized and converted into percentage scores out of 100 to facilitate analysis and interpretation. Internal consistency of the knowledge assessment was evaluated using Cronbach's alpha separately for each of the five knowledge domains and for the overall 38-item knowledge assessment.

## Data collection and analysis

After completion of data collection, survey responses were exported to Microsoft Excel for data cleaning, coding, and preparation for statistical analysis. The dataset was reviewed for completeness and consistency before analysis.

Categorical variables were summarized as frequencies and percentages. Knowledge scores were summarized using medians and interquartile ranges. Comparisons of knowledge scores between two groups were performed using the Mann–Whitney U test, while comparisons across more than two groups were performed using the Kruskal–Wallis test. When appropriate, *post hoc* pairwise comparisons were conducted using Bonferroni correction.

Spearman's rank correlation analysis was used to assess associations among the knowledge domain scores and the total knowledge score. Multivariable linear regression analysis was conducted to identify independent predictors of the total knowledge score, with results reported as unstandardized coefficients, 95% confidence intervals, and *p*-values. Multicollinearity among independent variables in the regression model was assessed using variance inflation factors (VIFs) and tolerance values.

All statistical tests were two-tailed, and a *p*-value of less than 0.05 was considered statistically significant. No imputation was performed for missing data; analyses were conducted using IBM SPSS Statistics for Windows, version 27.0 (IBM Corp., Armonk, NY, USA).

## Results

Of the 370 individuals invited to participate, 314 completed the questionnaire and were included in the analysis, yielding a response rate of 84.9%. Most respondents were younger than 40 years, including 125 participants aged 20–29 years (39.8%) and 100 aged 30–39 years (31.8%). Female participants comprised 60.8% of the sample, and nearly all respondents were Jordanian (97.8%).

Regarding academic and professional characteristics, 44.6% of participants held a bachelor's degree and 38.5% held a diploma. Technicians represented 62.1% of the sample, while radiographers accounted for 37.9%. Overall, 40.1% had ≤5 years of professional experience. Participants were employed mainly in private hospitals and centers (41.4%), followed by governmental (36.9%) and military facilities (21.7%). Although most participants reported the presence of a departmental policy for IV contrast media management (78.0%), 58.0% had not received any post-graduation training or knowledge update regarding contrast media, and only 33.4% had received specialized certification or training in managing contrast media reactions. In addition, 34.7% had previously observed a severe contrast media reaction during clinical practice. Self-reported confidence was most commonly moderate (34.7%), whereas 17.2% reported being very confident and 18.2% extremely confident in managing a severe IV contrast media reaction (Table 1).

**Table 1:** Participants’ demographic and professional characteristics.

Variable	Participants (*N* = 314), *n* (%)
Age	
20 −29 years 30–39 years 40–49 years 50–59 years > 60 years	125 (39.8%) 100 (31.8%) 56 (17.8%) 30 (9.6%) 3 (1.0%)
Gender	
Male Female	123 (39.2%) 191 (60.8%)
Nationality	
Non – Jordanian Jordanian	7 (2.2%) 307 (97.8%)
Education	
Diploma Bachelor Master PhD	121 (38.5%) 140 (44.6%) 39 (12.4%) 14 (4.5%)
Professional role	
Technician Radiographer	195 (62.1%) 119 (37.9%)
Duration of experience	
< 5 years 5–10 years 10–15 years 15–20 years 20–25 years > 25 years	126 (40.1%) 53 (16.9%) 53 (16.9%) 30 (9.6%) 17 (5.4%) 35 (11.1%)
Healthcare facility	
Governmental Private hospitals and centers Military	116 (36.9%) 130 (41.4%) 68 (21.7%)
Post-graduation training or knowledge update regarding contrast media	
Department Orientation Training Course Never learn after graduation	82 (26.1%) 50 (15.9%) 182 (58.0%)
Location of recent Academic Certification	
Outside Jordan Jordan	6 (1.9%) 308 (98.1%)
Departmental policy for IV contrast media management, yes	245 (78.0%)
Specialized certification/training in managing contrast media reactions, yes	105 (33.4%)
Observed a severe contrast media reaction, yes	109 (34.7%)
Confidence in the ability to manage a patient experiencing a severe reaction to IV contrast media	
Not at all confident Slightly confident Moderately confident Very confident Extremely confident	41 (13.1%) 53 (16.9%) 109 (34.7%) 54 (17.2%) 57 (18.2%)

Data are presented as *n* (%).

The median total knowledge score was 76.3% (IQR: 55.3–84.2). Among the individual domains, the highest median score was observed for general knowledge (100%, IQR: 80–100), followed by precaution knowledge (80%, IQR: 60–100), reaction recognition (80%, IQR: 50–80), and management knowledge (75%, IQR: 50–87.5). Risk factor knowledge showed the lowest median score (65%, IQR: 50–80) (Table 2).

**Table 2:** Domain and total knowledge scores among participants.

Variable	Participants (*N* = 314), median (IQR)
General knowledge score	100 (80–100)
Risk factor knowledge score	65 (50–80)
Precaution knowledge score	80 (60–100)
Reaction recognition score	80 (50–80)
Management knowledge score	75 (50–87.5)
Total knowledge score	76.3 (55.3–84.2)

Data are presented as median (interquartile range). All scores are expressed on a 0–100 scale.

Internal consistency analysis showed Cronbach's alpha coefficients of 0.804 for general knowledge, 0.707 for risk-factor knowledge, 0.659 for precaution knowledge, 0.642 for recognition of severe contrast media reactions, and 0.656 for management knowledge. The overall 38-item knowledge assessment demonstrated a Cronbach's alpha of 0.899.

Knowledge scores differed across educational levels in selected domains. Statistically significant differences were observed for risk factor knowledge (*p* = 0.019), reaction recognition (*p* = 0.004), and total knowledge score (*p* = 0.043). In contrast, general knowledge (*p* = 0.053), precaution knowledge (*p* = 0.935), and management knowledge (*p* = 0.772) did not differ significantly across educational groups (Table 3).

**Table 3:** Knowledge scores according to educational level.

Variable Median (IQR)	Diploma *N* = 121	Bachelor *N* = 140	Master *N* = 39	PhD *N* = 14	*P* value
General knowledge score	100 (80–100)	90 (60–100)	80 (60–100)	100 (80–100)	0.053
Risk factor knowledge score	70 (50–90)	60 (50–70)	60 (40–80)	50 (40–90)	0.019*
Precaution knowledge score	80 (60–100)	80 (60–100)	80 (40–100)	80 (80–100)	0.935
Reaction recognition score	80 (65–90)	70 (50–80)	70 (50–80)	60 (40–80)	0.004*
Management knowledge score	75 (62.5–87.5)	75 (50–87.5)	75 (62.5–75)	75 (62.5–87.5)	0.772
Total knowledge score	79 (60.5–84.2)	71 (52.6–84.2)	68.4 (50–81.6)	65.7 (57–89.5)	0.043*

Kruskal–Wallis test was used to compare scores across educational levels.

*p < 0.05 was considered statistically significant.

Knowledge scores differed significantly between technicians and radiographers in several domains. Radiographers had higher median scores than technicians for general knowledge (100% vs. 80%, *p* = 0.005), precaution knowledge (100% vs. 80%, *p* = 0.001), management knowledge (87.5% vs. 75.0%, *p* = 0.001), and total knowledge score (78.9% vs. 71.0%, *p* = 0.002). No statistically significant differences were observed between professional roles for risk factor knowledge or reaction recognition (Table 4).

**Table 4:** Knowledge scores according to professional role.

Variable	Technicians (*n* = 195), median (IQR)	Radiographers (*n* = 119), median (IQR)	*P* value
General knowledge score	80 (60–100)	100 (80–100)	0.005*
Risk factor knowledge score	60 (50–80)	70 (50–90)	0.112
Precaution knowledge score	80 (60–100)	100 (80–100)	0.001*
Reaction recognition score	70 (50–80)	80 (60–80)	0.660
Management knowledge score	75 (50–75)	87.5 (62.5–87.5)	0.001*
Total knowledge score	71 (52.6–81.6)	78.9 (63.1–86.8)	0.002*

Data are presented as median (interquartile range).

Scores are expressed on a 0–100 scale.

The Mann–Whitney U test was used for comparisons.

*p < 0.05 was considered statistically significant.

Knowledge scores were compared across categories of professional experience. Significant differences were observed for reaction recognition (*p* = 0.009) and management knowledge (*p* = 0.046), whereas general knowledge (*p* = 0.154), risk factor knowledge (*p* = 0.145), precaution knowledge (*p* = 0.141), and total knowledge score (*p* = 0.067) did not differ significantly across experience groups. Descriptively, the median reaction recognition score was 80 among participants with ≤5 years, 5–10 years, and >25 years of experience, compared with 70 among those with 10–15, 15–20, and 20–25 years of experience. For management knowledge, the median score ranged from 62.5 in the 20–25-year group to 87.5 in the >25-year group (Table 5).

**Table 5:** Knowledge scores according to duration of professional experience.

Variable	< 5 years *N* = 126	5–10 years *N* = 53	10–15 years *N* = 53	15–20 years *N* = 30	20–25 years *N* = 17	> 25 years *N* = 35	*P* value
General knowledge score	100 (80–100)	80 (60–100)	100 (60–100)	90 (60–100)	100 (60–100)	100 (60–100)	0.154
Risk factor knowledge score	65 (50–80)	60 (45–80)	70 (50–70)	60 (40–80)	60 (35–90)	90 (50–90)	0.145
Precaution knowledge score	80 (60–85)	80 (40–100)	80 (40–100)	80 (60–100)	80 (50–100)	80 (60–100)	0.141
Reaction recognition score	80 (60–90)	80 (50–80)	70 (40–80)	70 (50–80)	70 (55–75)	80 (60–80)	0.009*
Management knowledge score	75 (62.5–75)	75 (31–87.5)	75 (37–87.5)	75 (50–87.5)	62.5 (56–87.5)	87.5 (62.5–87.5)	0.046*
Total knowledge score	76 (62–81)	71 (48–86)	73 (46–84)	69.7 (55–84)	65.7 (55–89.5)	84 (55–89.5)	0.067

Data are presented as median (interquartile range).

The Kruskal–Wallis test was used to compare scores across professional experience categories.

Scores are expressed on a 0–100 scale.

*p < 0.05 was considered statistically significant.

**Figure 1:**
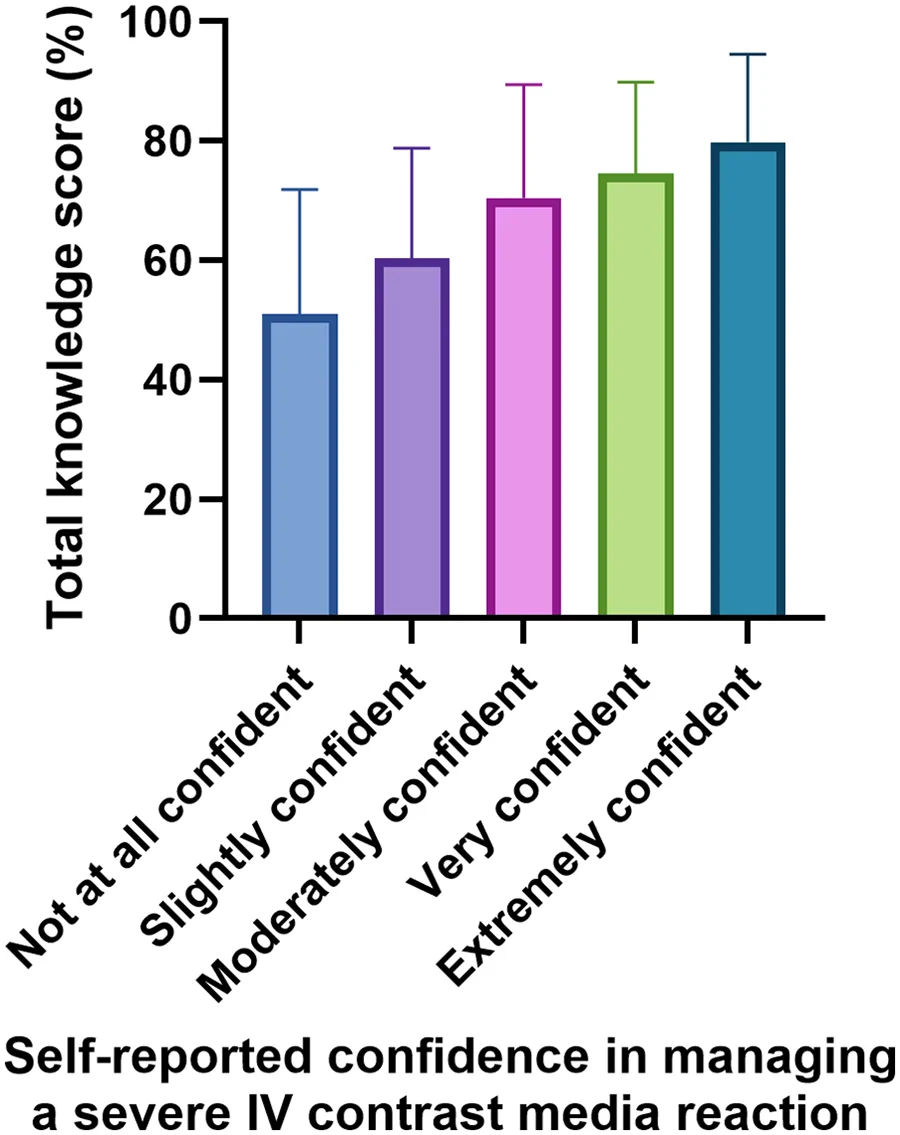
Total knowledge scores according to self-reported confidence in managing severe IV contrast media reactions.

Total knowledge scores increased across higher categories of self-reported confidence in managing severe IV contrast media reactions, as illustrated in Figure 1.

Spearman correlation analysis showed statistically significant positive associations among all knowledge domain scores (*p* < 0.01). The total knowledge score was strongly correlated with risk factor knowledge (*ρ* = 0.849), management knowledge (*ρ* = 0.817), precaution knowledge (*ρ* = 0.755), and reaction recognition (*ρ* = 0.748). Moderate correlations were observed between management knowledge and precaution knowledge (*ρ* = 0.632), management knowledge and reaction recognition (*ρ* = 0.585), and risk factor knowledge and management knowledge (*ρ* = 0.559) (Table 6).

**Table 6:** Spearman correlations among knowledge domain scores.

Variable	1	2	3	4	5	6
General knowledge score	1					
Risk factor knowledge score	0.458**	1				
Precaution knowledge score	0.490**	0.548**	1			
Reaction recognition score	0.476**	0.511**	0.450**	1		
Management knowledge score	0.513**	0.559**	0.632**	0.585**	1	
Total knowledge score	0.664**	0.849**	0.755**	0.748**	0.817**	1

Values are Spearman's rank correlation coefficients (*ρ*). **p < 0.01.

In the multivariable linear regression model, several variables were independently associated with total knowledge score. Compared with participants aged 20–29 years, those aged 30–39 years had lower total knowledge scores (B = −6.4, *p* = 0.035). Male participants had lower scores than female participants (*B* = −7.6, *p* = 0.001). Compared with diploma holders, participants with a master's degree had higher scores (*B* = 8.5, *p* = 0.021). Radiographers also had higher total knowledge scores than technicians (*B* = 5.6, *p* = 0.016). Participants with >25 years of experience had lower scores than those with ≤5 years of experience (*B* = −10.5, *p* = 0.027). Higher self-reported confidence in managing severe IV contrast media reactions was progressively associated with higher total knowledge scores, with coefficients ranging from *B* = 12.4 among slightly confident participants to *B* = 26.6 among extremely confident participants. Nationality, healthcare facility type, location of recent academic or professional certification, prior training or knowledge update, departmental policy, specialized certification or training, and previous observation of a severe contrast media reaction were not independently associated with total knowledge score. Assessment of multicollinearity showed VIF values ranging from 1.12 to 3.17 (mean VIF = 1.89), with tolerance values ranging from 0.315 to 0.895, indicating no evidence of problematic multicollinearity. The model explained 37.0% of the variance in total knowledge score (R^2^ = 0.370; adjusted R^2^ = 0.310) (Table 7).

**Table 7:** Multivariable linear regression analysis of factors associated with total knowledge score.

Variable	B coefficient	95% CI	*P* value
Age			
20 −29 years 30–39 years 40–49 years 50–59 years > 60 years	ref −6.4 −1.7 6.4 8	(−12.4 - −0.5) (−10.1 - 6.7) (−3.0 - 15.9) (−12.6 - 28.7)	- 0.035* 0.694 0.179 0.445
Nationality			
Non – Jordanian Jordanian	ref 12.3	(−1.3 - 25.9)	- 0.077
Sex			
Male Female	−7.6 Ref	(−11.6 - −3.7)	0.001*
Education			
Diploma Bachelor Master PhD	ref −3.2 8.5 −0.54	(−8.2 - 1.8) (1.3 - 15.8) (−10.7 - 9.6)	- 0.207 0.021* 0.916
Professional role			
Technician Radiographer	ref 5.6	(1.1 - 10.2)	- 0.016*
Duration of experience			
< 5 years 5–10 years 10–15 years 15–20 years 20–25 years > 25 years	ref -3.7 -3.7 -4.2 -9.6 -10.5	(−10.2 - 2.7) (−15.1 - 0.6) (−13.1 - 4.7) (−20.6 - 1.3) (−19.8 - −1.2)	- 0.235 0.071 0.350 0.084 0.027*
Healthcare facility type			
Governmental Private hospitals and centers Military	ref 2.9 -3.4	(−1.9 - 7.8) (−9.1 - 2.3)	- 0.236 0.239
Training or knowledge update regarding contrast media			
Department Orientation Training Course Never learn after graduation	Ref -2.2 5.13	(−8.5 - 4.2) (−0.3 - 10.6)	- 0.500 0.065
Location of recent academic/professional certification			
Outside Jordan Jordan	ref 10.4	(−4.0 - 24.8)	- 0.156
Departmental policy for IV contrast media management, yes	4.2	(−0.9 - 9.3)	0.106
Specialized certification/training in managing contrast media reactions, yes	-2.7	(−7.4 - 2.0)	0.253
Previous observation of a severe contrast media reaction, yes	1.6	(−3.3 - 6.4)	0.525
Self-reported confidence in managing severe IV contrast media reactions			
Not at all confident Slightly confident Moderately confident Very confident Extremely confident	Ref 12.4 18.4 20.4 26.6	(5.1 - 19.7) (11.9 - 25.0 (12.9 - 27.9) (18.1–35.1)	- 0.001* <0.001* <0.001* <0.001*

B, unstandardized regression coefficient; CI, confidence interval. Female gender, age 20–29 years, non-Jordanian nationality, diploma education, technician role, ≤5 years of experience, governmental facility, department orientation, outside-Jordan certification, no departmental policy, no specialized certification/training, no previous severe reaction observation, and “not at all confident” were used as reference categories. R^2^ = 0.370; adjusted R^2^ = 0.310. VIFs ranged from 1.12 to 3.17 (mean VIF = 1.89), indicating no evidence of problematic multicollinearity. *p* < 0.05 was considered statistically significant.

## Discussion

This study evaluated knowledge of severe contrast media reactions among CT technicians and radiographers in Jordan across five domains: general knowledge, risk factor knowledge, precaution knowledge, recognition of severe reactions, and management knowledge. Overall, participants demonstrated a moderate-to-high total knowledge score, although performance varied across domains. General knowledge showed the highest median score, whereas risk factor knowledge was the lowest-performing domain. This finding suggests that while participants were generally familiar with basic concepts related to contrast media, important gaps remain in identifying patients at increased risk of adverse reactions. Such gaps are clinically relevant because risk recognition is central to prevention, preparedness, and timely escalation before and during contrast-enhanced CT examinations (8, 9, 12, 15).

The relatively lower score in risk factor knowledge is particularly important in the context of international recommendations, which emphasize patient assessment, recognition of previous contrast reactions, asthma, renal impairment, cardiovascular comorbidity, medication-related risk, and readiness for emergency management (8, 9, 12, 16). Although severe reactions to modern iodinated contrast media are uncommon, their potential seriousness requires radiology staff to be prepared for rapid recognition and response (4, 5, 15). Therefore, knowledge deficits in risk stratification may reduce the effectiveness of preventive measures and delay appropriate escalation in high-risk patients.

Professional role was significantly associated with several knowledge domains. Radiographers had higher scores than technicians in general knowledge, precaution knowledge, management knowledge, and total knowledge score. This difference may reflect variation in educational preparation, professional scope, clinical exposure, and role-specific responsibilities during contrast-enhanced imaging procedures (6, 14). Radiographers may receive more structured training in imaging protocols, patient safety, contrast administration, and emergency response than technicians. However, because this study used a cross-sectional design, these findings should be interpreted as associations rather than evidence that professional role directly causes higher knowledge.

These findings are broadly consistent with previous research showing that preparedness for contrast media reactions varies across radiology professionals and healthcare settings. Hadi et al. reported significant variation in knowledge of severe contrast media reactions among CT radiographers, with professional seniority and confidence-related factors contributing to differences in preparedness (6). Similarly, studies from other settings have shown that although radiographers may be aware of basic precautions and common reaction patterns, gaps often persist in comprehensive knowledge, emergency management, and practical readiness (7, 17–19). Together, these findings indicate that contrast media safety cannot rely only on routine clinical exposure but requires structured, recurrent, and role-specific education.

Educational level was also associated with selected knowledge domains. Significant differences were observed for risk factor knowledge, reaction recognition, and total knowledge score. In the regression model, holding a master's degree was independently associated with a higher total knowledge score compared with diploma-level education. This may reflect greater exposure to advanced clinical reasoning, evidence-based practice, and patient safety principles among participants with postgraduate education. However, the absence of consistent significant differences across all domains suggests that formal academic qualifications alone may not ensure competence in contrast reaction management. Targeted training focused on practical emergency response remains necessary regardless of educational level (6, 8, 9).

Professional experience showed a more complex pattern. Reaction recognition and management knowledge differed across experience groups, but total knowledge score was not significantly different across experience categories. In the regression model, participants with more than 25 years of experience had lower total knowledge scores than those with ≤5 years of experience. This finding should be interpreted cautiously, as it may reflect differences in recent training exposure, changes in educational curricula, or reduced access to updated contrast media protocols among more experienced staff. It also highlights that experience alone may not be sufficient to maintain current knowledge, particularly in areas where guidelines and safety practices continue to evolve (6, 7). These findings partially align with our previous Jordanian study, in which knowledge of CT exposure parameters varied according to educational level, professional role, and years of experience (20).

Self-reported confidence was strongly associated with total knowledge score. Participants with higher confidence in managing severe IV contrast media reactions had higher total knowledge scores, and this association remained evident in the multivariable regression model. This association indicates that perceived readiness may partly reflect actual knowledge; however, confidence should not be considered a substitute for objective competence assessment. Previous research has similarly shown that confidence in managing contrast media reactions is linked to knowledge and preparedness, but studies have also reported mismatches between perceived confidence and actual performance (6, 7, 17). Therefore, training programs should aim to improve both knowledge and practical confidence through simulation, emergency drills, and competency-based assessment (21, 22).

An important finding was the discrepancy between the reported availability of departmental policies and the limited formal training reported by participants. Although most participants indicated that their department had a policy for IV contrast media management, more than half had not received post-graduation training or knowledge updates, and only one-third had received specialized training in managing contrast media reactions. Moreover, prior training, specialized certification, and departmental policy were not independently associated with total knowledge score in the regression model. In contrast, our previous Jordanian study found that attendance at specialized CT training courses was associated with better knowledge performance regarding CT exposure parameters (20). This may indicate variability in the quality, recency, content, or practical emphasis of existing training programs and policies. Written policies alone may be insufficient unless they are accompanied by regular education, practical rehearsal, and clear role allocation during emergencies (6, 8, 9, 12).

The significant positive correlations among all knowledge domains further suggest that competence in severe contrast media reaction management is multidimensional. Higher total knowledge scores were strongly correlated with risk factor knowledge, management knowledge, precaution knowledge, and reaction recognition. This pattern supports the need for integrated training programs that address the full pathway of contrast media safety, from pre-procedure risk assessment to recognition of severe reactions, immediate management, documentation, and escalation of care (8, 9). Training that focuses only on isolated facts may be less effective than scenario-based programs that link risk identification, clinical decision-making, and emergency response (21, 22).

Simulation-based education may be particularly valuable in this context. Severe contrast media reactions are relatively rare, meaning many radiology professionals may have limited real-life exposure to these events (4, 5). High-fidelity simulation, standardized emergency checklists, and multidisciplinary drills involving radiographers, technicians, radiologists, nurses, and emergency response teams may improve both knowledge and response readiness. Previous work has shown that simulation-based contrast reaction training can improve comfort and preparedness, while standardized checklists may reduce critical management errors during emergencies (21, 22). Incorporating such approaches into continuing professional development may help strengthen patient safety in contrast-enhanced CT practice.

This study has several practical implications. Radiology departments should provide regular, structured training on severe contrast media reactions for both CT technicians and radiographers. Training should prioritize risk factor recognition, early identification of severe reactions, emergency medication awareness, airway and oxygen support principles, rapid response activation, and documentation (8, 9, 12, 15). Departments should also ensure that contrast reaction protocols are not only available but actively implemented through periodic drills and competency assessments. Given the observed differences between technicians and radiographers, educational programs should be tailored to the responsibilities and baseline knowledge of each professional group (6, 7, 14).

## Limitations

This study has some limitations. Its cross-sectional design prevents causal inference between professional characteristics, confidence, training, and knowledge scores. The use of self-reported data may introduce recall or social desirability bias, particularly for prior training, observed reactions, and confidence. The study assessed knowledge using a questionnaire rather than direct observation of clinical performance during simulated or real emergencies. In addition, the sample was predominantly Jordanian and may not fully represent radiology professionals in other healthcare systems. No formal *a priori* sample-size calculation or power analysis was performed, as recruitment was based on feasibility and accessibility of eligible participants during the study period which should be considered when interpreting the precision and generalizability of the findings. Despite these limitations, the study provides important evidence regarding knowledge gaps and training needs among CT technicians and radiographers involved in contrast-enhanced imaging.

## Conclusion

In conclusion, CT technicians and radiographers in Jordan demonstrated moderate-to-high overall knowledge of severe contrast media reactions, but important gaps were observed, particularly in risk factor knowledge. Radiographers generally achieved higher knowledge scores than technicians, and higher self-reported confidence was associated with higher total knowledge scores. The findings highlight the need for structured, recurrent, and competency-based training programs that integrate risk assessment, reaction recognition, and emergency management. Simulation-based education, standardized checklists, and multidisciplinary response protocols may help improve preparedness and patient safety during contrast-enhanced CT procedures.

## Data Availability

The original contributions presented in the study are included in the article/Supplementary Material, further inquiries can be directed to the corresponding author.
